# Limp in a Child With Autism Spectrum Disorder

**DOI:** 10.1177/2333794X17744139

**Published:** 2017-11-30

**Authors:** Adam Yan, Melanie Conway, Carolyn E. Beck

**Affiliations:** 1 The Hospital for Sick Children, Toronto, Ontario, Canada; 2 University of Toronto, Toronto, Ontario, Canada

## Case

A 6-year-old nonverbal male with autism spectrum disorder (ASD) presented to a community hospital’s emergency department with a 2-week history of limp and oral mucosal bleeding. At the time, he was dragging his left leg and refusing to bear weight. Despite no clear history of injury, he was evaluated for a potential traumatic cause for his limp. Plain films of both hips and legs were obtained and revealed no abnormalities. Initial blood work showed a hemoglobin of 90 g/L (normal = 120-160 g/L), mean corpuscular volume of 64 fL (normal = 77-96 fL), leukocytes of 7.4 × 10^9^/L (normal = 4-10 × 10^9^/L), and platelets of 271 × 10^9^/L (normal = 150-400 × 10^9^/L). Given a maternal history of rheumatoid arthritis, an antinuclear antibody and rheumatoid factor were sent. No explanation for the limp was found, and the child was discharged home with a referral to dentistry to address the oral mucosal changes.

The child then presented to our pediatric hospital with new onset of fevers for 1 week, ongoing limp that had progressed to complete refusal to weight bear, and persistent bleeding from his oral mucosa. He was evaluated in the emergency department, and a repeat X-ray of his left leg showed swelling at the lateral distal femur concerning for an early infection and mild osteopenia. Blood work revealed a hemoglobin of 83 g/L, mean corpuscular volume of 63 fL, and platelets of 320 × 10^9^/L. The antinuclear antibody and rheumatoid factor previously sent were both negative. Dentistry evaluated the child and found no dental abnormality. Given his radiographic findings, fever, and limp, the patient was admitted to general pediatrics to rule out osteomyelitis. Intravenous cefazolin was commenced, and a magnetic resonance imaging (MRI) scan was requested.

## Hospital Course

On evaluation by the inpatient pediatric team, he was found to have hyperkeratosis of his hands, significant gum hyperplasia, halitosis, and mucosal bleeding. On examination of his left leg, there was no point tenderness or erythema, but there was some mild edema of his left knee, and he was significantly distressed by any manipulation of the limb. Dietary history revealed a very restrictive diet comprising only cereal and milk with occasional scrambled eggs. The inpatient team deferred the MRI and discontinued the intravenous antibiotics. A panel of nutritional markers was sent, and a presumptive diagnosis of scurvy was made. The patient was started on vitamin C 250 mg daily, an iron supplement, and a multivitamin. The child was discharged home with general pediatrics and dietician follow-up. Subsequent results showed an ascorbic acid (vitamin C) level <5 µmol/L (normal = >24 µmol/L), a ferritin of 31.1 µg/L (normal = 13.7-78.8 µg/L), total 25-OH vitamin D level of 99 nmol/L (normal = 70-250 nmol/L), a red cell folate of 464 nmol/L (normal = 182-834 nmol/L), and a normal hemoglobin analysis. The low ascorbic acid level confirmed the diagnosis of scurvy with a concomitant diagnosis of anemia.

At follow-up, 2 months postdischarge, the child had regained full ability to ambulate and was no longer having mucosal bleeding.

## Final Diagnosis

Scurvy

## Discussion

Traditionally, one would associate scurvy with “Sailors at Sea,” who traveled on prolonged voyages consuming diets deficient in vitamin C. However, in modern days, scurvy is increasingly identified in children with ASD and developmental delay who consume restrictive diets, often lacking in fruits and vegetables.^1^ The recommended daily allowance of vitamin C is 15 to 45 mg for children aged 1 to 13 years and 65 to 75 mg for children aged 14 to 18 years.^2^ Overt symptoms of scurvy can begin to develop as early as 3 months after deficient intake begins.^3^

The presentation of scurvy can be variable. Early features of scurvy may be nonspecific and include leg pain, irritability, decreased oral intake, and fevers. More severe cases can present with multisystem disease with dermatologic, mucosal, and musculoskeletal findings. Dermatologic findings can include perifollicular hemorrhages, petechiae, purpura, ecchymosis, hyperkeratosis, and corkscrew hairs. Mucosal manifestations may include gingival hyperplasia, gingival bleeding, and halitosis. Typical musculoskeletal complaints include bone pain, arthritis, edema, fractures, a depressed sternum, and subperiosteal hemorrhages.^4^ Although imaging is not required for diagnosis, it is often part of the initial workup for musculoskeletal pain. The most common associated radiographic sign is osteopenia, but more specific signs include the following: Pelkan spurs from healing metaphyseal fractures, Wimberger ring sign, and periosteal bone formation secondary to subperiosteal hemorrhage.^5^

Clinical signs are the first clue to the diagnosis of scurvy; however, confirmation is required with laboratory tests. A low plasma level of vitamin C <0.2 mg/dL (<17.7 µmol/L) is usually considered deficient and is highly specific for the diagnosis of scurvy. The plasma vitamin C level is not a sensitive diagnostic test for scurvy because it is readily affected by recent vitamin C consumption.^2^ A better estimate of the body’s store of vitamin C can be obtained through the measurement of the vitamin C level in the buffy-coat of leukocytes. Unfortunately, obtaining leukocyte buffy-coat vitamin C levels is technically difficult and not readily available. An alternative method of assessing body stores of vitamin C is to measure urinary excretion of vitamin C after an ascorbic acid infusion.^3^

A heterogeneous group of children with a variety of medical conditions are at risk of developing scurvy. This includes patients suffering from iron overload because of multiple transfusions in the context of sickle cell disease or thalassemia, bone marrow transplant recipients, patients on chemotherapy, and those with diets deficient in vitamin C.^5^ Diets devoid of vitamin C can be seen in the neonatal period in children consuming pasteurized or boiled animal milk, or on prolonged total parenteral nutrition. In older children, dietary deficiency of vitamin C is commonly seen in those with underlying neurodevelopmental disorders such as ASD.

ASD is a neurodevelopmental disorder affecting 1% to 2% of the population, characterized by deficits in social behavior and nonverbal interactions, and sensory processing abnormalities.^6^ Due to the unique way in which children with ASD process sensory information, food selectivity can be a major struggle.^7^ In ASD, up to 90% of patients report a restrictive dietary pattern that puts them at risk of developing scurvy.^8^ One case series at a single American pediatric hospital found 7 children in an 18-year time period with ASD and scurvy.^9^

Our case illustrates a classic presentation of scurvy, with manifestations that directly reflect the crucial role of ascorbic acid in collagen synthesis and cross-linking.^10^ Specifically, a lack of ascorbic acid leads to blood vessel instability from pericapillary collagen depletion.^11^ This accounts for the patient’s gingival bleeding, as well as his left leg pain secondary to subperiosteal hemorrhage. The anemia in scurvy is multifactorial, because of a combination of inadequate nutritional intake, blood loss, decreased iron absorption, and inflammation.

## Conclusion

This case serves as a reminder that even in an age where high-resolution imaging is readily available at our fingertips, one must not underestimate the importance of taking a detailed dietary history. In doing so, our patient was spared from invasive investigations including an MRI and the associated risks of a general anesthetic. Clinicians should maintain a high index of suspicion for nutritional deficiencies in patients with ASD. The high prevalence of nutritional deficiencies suggests that perhaps there is a role for screening for vitamin C deficiency and other nutritional deficiencies in at-risk populations and in children with unexplained anemia. Earlier identification of this child’s restrictive diet could have allowed for health care providers to intervene prior to the development of severe clinical manifestations of the disease.
